# Differential susceptibility of retinal ganglion cell subtypes in acute and chronic models of injury and disease

**DOI:** 10.1038/s41598-020-71460-6

**Published:** 2020-10-15

**Authors:** Kirstin B. VanderWall, Bin Lu, Jorge S. Alfaro, Anna R. Allsop, Alexa S. Carr, Shaomei Wang, Jason S. Meyer

**Affiliations:** 1 Department of Biology, Indiana University Purdue University Indianapolis, Indianapolis, IN 46202 USA; 2 Department of Biomedical Sciences, Cedars-Sinai Medical Center, Regenerative Medicine Institute, Los Angeles, CA 90048 USA; 3 Department of Medical and Molecular Genetics, Indiana University School of Medicine, Indianapolis, IN 46202 USA; 4 Deparment of Ophthalmology, Glick Eye Institute, Indiana University School of Medicine, Indianapolis, IN 46202 USA; 5 Stark Neurosciences Research Institute, Indiana University School of Medicine, Indianapolis, IN 46202 USA

**Keywords:** Retina, Cell death in the nervous system

## Abstract

Retinal ganglion cells (RGCs) are a heterogeneous population of neurons, comprised of numerous subtypes that work synchronously to transmit visual information to the brain. In blinding disorders such as glaucoma, RGCs are the main cell type to degenerate and lead to loss of vision. Previous studies have identified and characterized a variety of RGC subtypes in animal models, although only a handful of studies demonstrate the differential loss of these RGC subtypes in response to disease or injury. Thus, efforts of the current study utilized both chronic (bead occlusion) and acute (optic nerve crush, ONC) rat models to characterize disease response and differential loss of RGC subtypes. Bead occlusion and ONC retinas demonstrated significant RGC loss, glial reactivity and apoptosis compared to control retinas. Importantly, bead occlusion and ONC retinas resulted in differential subtype-specific loss of RGCs, with a high susceptibility for alpha- and direction selective-RGCs and preferential survival of ipRGCs. Results of this study serve as an important foundation for future experiments focused on the mechanisms resulting in the loss of RGCs in optic neuropathies, as well as the development of targeted therapeutics for RGC subtype-specific neuroprotection.

## Introduction

Retinal ganglion cells (RGCs) are the only projection neurons of the retina, extending axons through the optic nerve to connect with post-synaptic targets in the brain^1^. Disruptions to this crucial connection due to injury or disease result in the degeneration of RGCs and subsequent loss of vision or blindness^2–5^. Although most RGCs function to transmit visual information to the brain, this class of cells has been proven to be highly diverse, with the classification of more than 40 subtypes to date^6–9^. RGC subtypes have been previously classified based on differences in morphological features and functional capabilities, collectively contributing to the multifaceted integration of visual stimuli. In more recent years, the identification of RGC subtypes has been achieved through the expression of specific molecular signatures in a variety of animal models^7,8,10,11^, with this ability to successfully identify RGC subtypes allowing for more in-depth studies of their development and contribution to vision. Furthermore, the ability to recognize how various RGC subtypes are susceptible to disease or injury allows for the analysis of disease mechanisms and the development of targeted treatments.

Previous studies have demonstrated the identification and subsequent degeneration and death of RGC subtypes in variety of injury and disease models^12–25^, although very few have utilized the more recently developed elevated intraocular pressure (IOP) bead occlusion model of glaucoma^26–30^ for these purposes. Additional studies depicting the precise loss of RGC subtypes in multiple representative models of glaucoma would provide necessary insight into the development of specific therapeutics.

Thus, efforts of this study utilized a chronic bead occlusion model of elevated IOP, as well as an acute ONC injury model, to analyze RGC loss and disease-related phenotypes. Bead injected and ONC retinas resulted in a significant loss of RGCs correlated with retinal thinning, glial reactivity, and increased apoptosis. When specific RGC subtypes were examined following bead injection or ONC, direction selective-RGCs and alpha-RGCs were found to be highly susceptible to injury, while non-image forming intrinsically photosensitive-RGCs (ipRGCs) were found to be highly resilient. The results of this study are among the first to utilize the chronic elevated-IOP bead occlusion model of glaucoma for studying differential RGC subtype loss and provide additional insights into the loss of RGCs following injury. More so, these results will aid in the development of therapeutics targeted at specific subtypes, with important implications for the neuroprotection of those subtypes lost in optic neuropathies.

## Methods

### Establishment of animal models

For ONC models, Long-Evans rats (n = 9) were anesthetized via intraperitoneal (IP) injection of anesthetic agents, followed by subcutaneous injection of carprofen. In each animal, only one eye was subjected to experimental operation, while the fellow eye was used as an untreated control. Animal heads were fixed in a headholder with intraorbital optic nerves exposed after a lateral canthotomy and limbal peritomy. Right optic nerves (ON) were surgically exposed in their intraorbital segments, spanning 2 to 3 mm beyond the eye cup, with care taken to leave the retinal vascularization intact. The lacrimal gland was displaced, but left intact, and the superior extraocular muscles were spread to allow access to the ON. An incision was made in the eye-retractor muscle and the meninges perpendicular to the axonal orientation, extending over one third of the dorsal ON. The ON was then pinched with forceps at a distance of 1 mm behind the eye, leaving the retinal vascularization unaffected. All microsurgeries were performed with the aid of a surgical microscope. Post-surgical procedures consisted of a reversal agent via IP, and topical application of ophthaine and vetropolycin before placing rats under a heat lamp until confirmed recovery from anesthesia.

To elevate the intraocular pressure (IOP) for the chronic bead occlusion model, a modified protocol was followed according to a published study^30^. Briefly, an intracameral injection of a 2.5% polystyrene microbead suspension was used, consisting of microbeads with a particle diameters of 10.0 µm and 6.0 µm (Polysciences, Warrington, PA, US) in phosphate buffered saline (PBS). Sprague Dawley rats (n = 9) were anesthetized, one eye was operated, and the contralateral eye was used as control. A glass microneedle with a tip diameter of approximately 50 µm was connected with a syringe via plastic tubing. The microneedle was loaded with 5ul of microbeads with a particle diameter of 10.0 µm followed by 5 µl of microbeads with a particle diameter of 6.0 µm. This arrangement enabled the injection of the smaller beads first, followed by the larger beads. Before loading the microneedle, the beads were washed in absolute ethanol and centrifuged for one minute at 1000 rpm, and then washed three times in PBS. For injection, a glass microneedle was injected into the anterior chamber near the corneal limbus at an angle of about 45 degrees to the lower quadrant corneal surface, avoiding contact with lens. The total volume of bead suspension was injected rapidly, and the needle was kept in place for 3 min before pulling out to reduce efflux of beads. Subsequently, IOP was measured at several time points after surgery. Both operated and untreated eyes were measured with a laboratory rebound tonometer (TonoLab, Icare, Finland). An average of three readings was recorded for each eye. At the end of each experiment, both experimental eyes as well as contralateral control eyes were collected and processed for cryostat sections.

The institutional animal care and use committee of Cedars-Sinai Medical Center approved the animal procedures performed in this study and these procedures were performed in compliance with the Association for Research in Vision and Ophthalmology Statement for the Use of Animals in Ophthalmic and Vision Research. Experimental protocols were also performed in accordance and approved by the Cedars-Sinai Institutional Biosafety Committee.

### Histological analysis of retinal tissue

To analyze retinas by immunocytochemistry, eyeballs were enucleated and fixed in 4% paraformaldehyde. Retinal tissue was dissected away from the rest of the eye and placed on an embedding mold, with retinal sections cut from superior-dorsal to inferior-ventral (Supplemental Fig. 1). 10 µm sections were collected in five series, with 4 sections per slide and each section contained nasal and temporal regions. Retinal sections were permeabilized with 0.2% Triton-X-100 in PBS for 10 min at room temperature, followed by a wash with 1 × PBS. Slides were then blocked using 10% Donkey Serum in PBS for 1 h at room temperature. Primary antibodies were prepared in 0.1% Triton-X-100 and 5% Donkey Serum and applied to the slides overnight at 4 °C (Supplemental Table 1). The following day, the primary antibodies were removed from the slides and washed three times with 1X PBS. Samples were then blocked with 10% Donkey Serum for 10 min at room temperature. Secondary antibodies were prepared in 0.1% Triton-X-100 and 5% Donkey Serum and applied to the slides for 1 h at room temperature in the dark. Secondary antibodies were washed three times in 1X PBS and then mounted using cover glass and poly-mount mounting medium.

**Figure 1:**
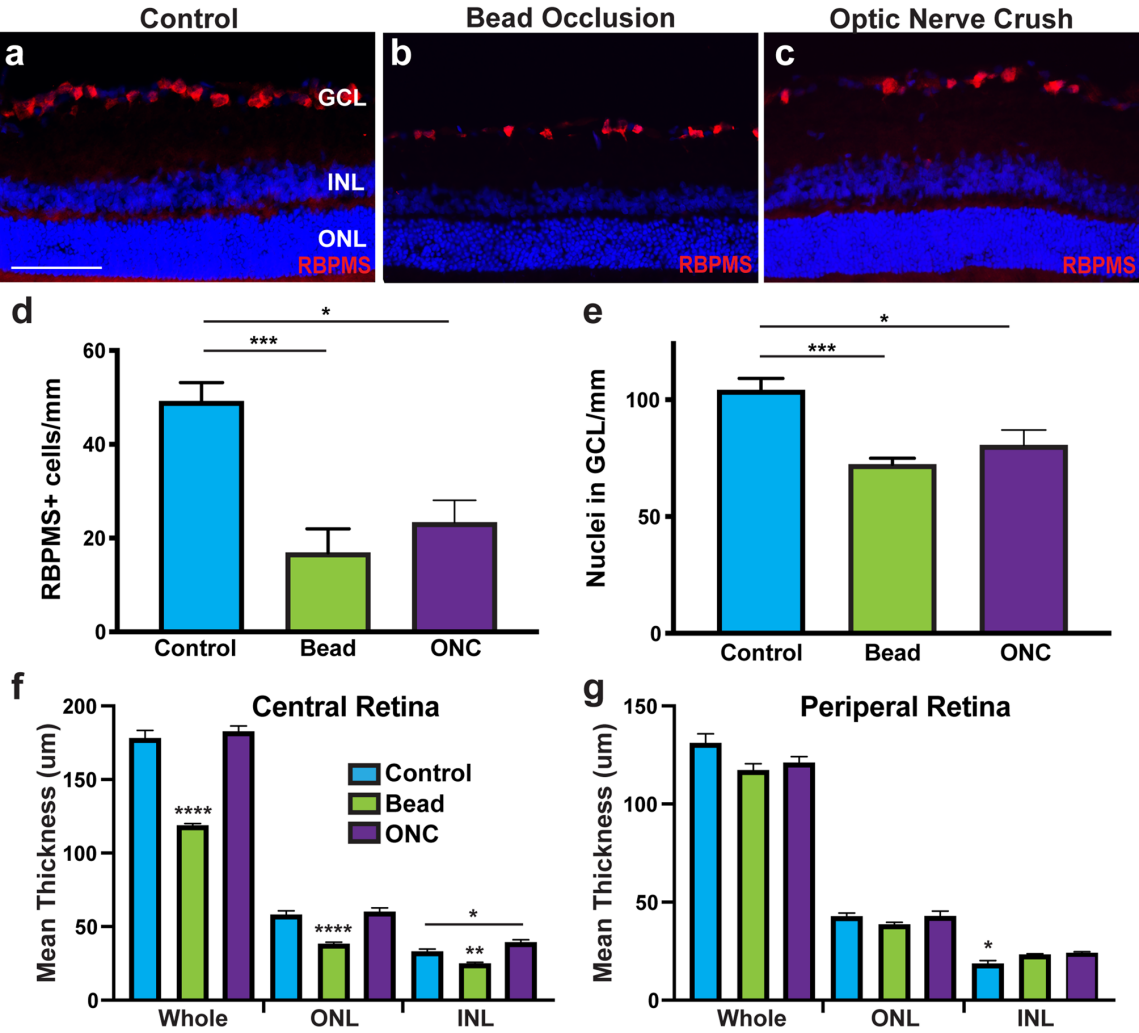
Chronic bead injection and acute ONC injuries result in profound RGC loss and retinal thinning. (**a**–**d**). RBPMS expression was identified within the RGC layer, with the average number of RBPMS-expressing RGCs significantly decreased following bead injection and ONC compared to controls. (**e**) Bead injected and ONC retinas also demonstrated a significant decrease in the number of DAPI-positive nuclei within the RGC layer compared to controls. Error bars represent S.E.M. Scale bars equal 100 µm. Statistical differences were determined using a One-way ANOVA followed by Tukey’s post hoc with 95% confidence. (*p < 0.05, ***p < 0.001). n = 6 retinas were analyzed per group, with 4 cross sections and numerous technical replicates per cross section. Cross sections contained both nasal and temporal parts, with technical replicates including areas from both the central and peripheral retina.

### Quantification and statistical analyses

Pan-RGC and RGC subtype-specific antibodies were quantified from n > 3 biological replicates and at least 4 technical replicates per biological replicate. The expression of each marker was quantified with ImageJ software using the cell counter plugin, and retinal length and thickness were calculated using appropriate scaling in ImageJ. Fluorescent intensities were calculated using ImageJ plugins by identifying a region of interest (ROI) within inner most layers, thresholding intensity over background fluorescence and measuring mean gray value / ROI area. For these calculations, fluorescent intensities were kept consistent among replicates. Statistical differences were calculated using GraphPad Prism software by a student’s t-test or a One-Way Anova with Tukey’s post-hoc excluding outliers and using a 95% confidence interval.

## Results

### Chronic and acute models result in RGC loss and disease phenotypes

The degeneration of RGCs is commonly associated with a number of other phenotypes including significant loss of RGCs, glial reactivity, inflammatory responses, and increased apoptosis^3–5^. Therefore, initial efforts were aimed at examining these disease phenotypes following chronic (bead injection) or acute (ONC) injury models (Fig. 1). Bead injected animals were evaluated for elevated IOP at 1, 4, and 5 weeks after injection and demonstrated a significant increase in IOP at 1 and 4 weeks (Supplementary Fig. 2). Immunocytochemistry for the pan-RGC marker RBPMS demonstrated a significant decrease in the average number of RGCs within the ganglion cell layer in bead injected eyes or after ONC (Fig. 1a–e). In glaucomatous injury models, the number of DAPI-positive nuclei were also significant decreased in the ganglion cell layer compared to control retinas. Lastly, the thickness of the retina was measured in central and peripheral retinal sections, with bead injected rats demonstrating significantly thinner retinal layers compared to control or ONC (Fig. 1f–g). Overall, these results validated the utilization of the bead occlusion and ONC models as chronic and acute models, respectively, for the degeneration of RGCs observed in glaucoma.

**Figure 2:**
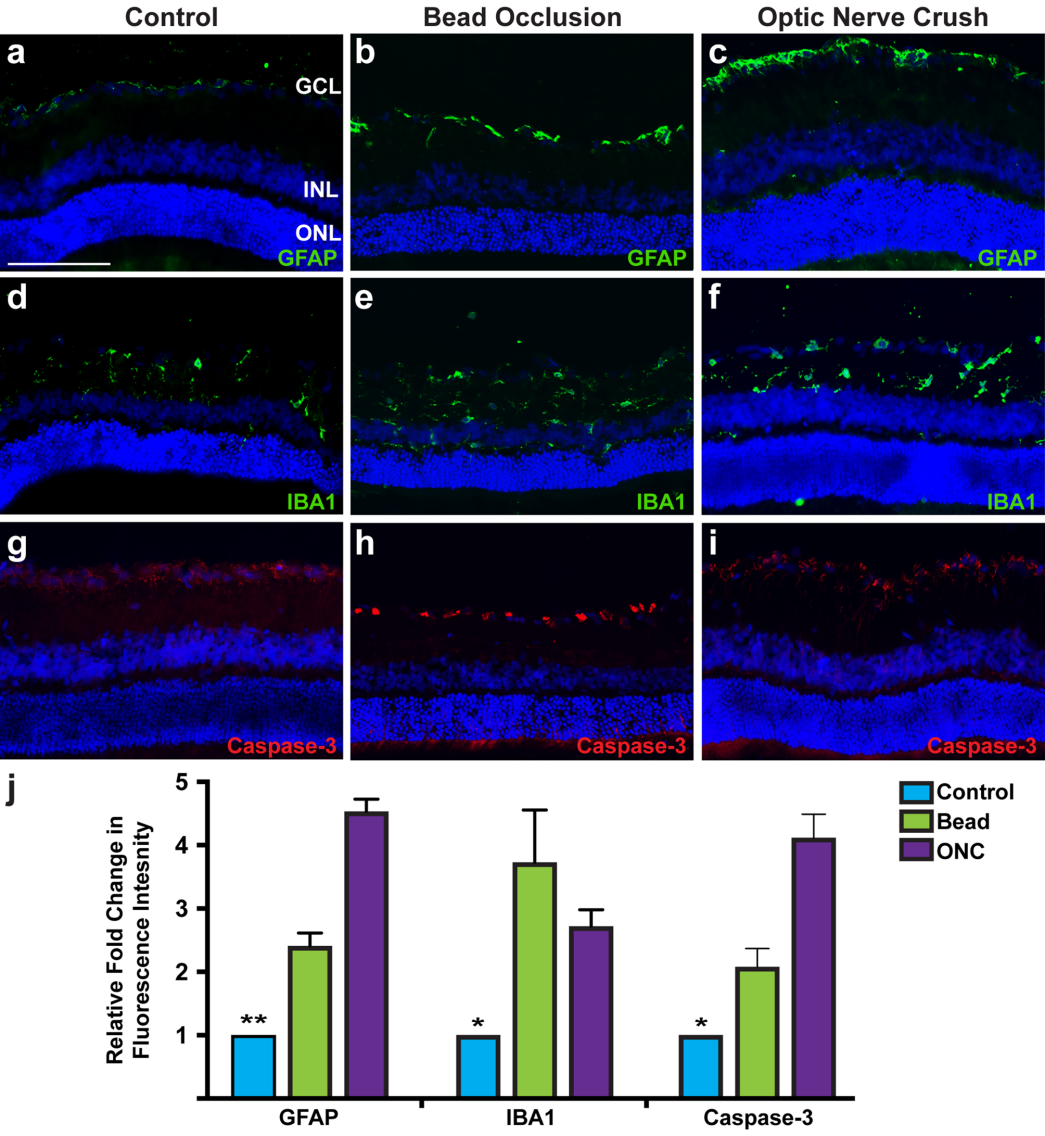
Glaucomatous injury leads to glial activation and apoptosis. (**a**–**c**, **j**) Expression of GFAP was displayed in close association with RGCs in the nerve fiber layer, with significantly higher intensity in bead injected and ONC retinas. (**d**–**f**, **j**) Microglia were identified throughout layers inward of the ONL by IBA1, with increased intensity of microglia and morphological changes of these cells in bead injected and ONC retinas. (**g**–**j**) Increased apoptosis was observed after bead injection and ONC through increased expression of active caspase-3 compared to controls. Error bars represent S.E.M. Scale bars equal 100 µm. Statistical differences were determined using a One-way ANOVA followed by Tukey’s post hoc with 95% confidence. (*p < 0.05, **p < 0.01). n = 6 retinas were analyzed per group, with 4 cross sections and numerous technical replicates per cross section. Cross sections contained both nasal and temporal parts, with technical replicates including areas from both the central and peripheral retina.

Subsequently, retinas were analyzed for other phenotypes typically associated with injury and disease^31–34^. Glial activation has been previously demonstrated to be associated with disease and injury^35–38^. This gliosis normally functions as a safety mechanism in response to an assault, attempting to halt any further damage to the affected area, as well as provide support to the area in order to heal damage that has occurred. In both bead injected and ONC injury models, retinas exhibited significant upregulation of GFAP within the nerve fiber layer, characteristic of astrocyte reactivity (Fig. 2). Additionally, compared to the controls, bead injected and ONC animals demonstrated increased microglia infiltration based on the expression of IBA1. Apoptotic activation was examined by the expression of active caspase-3, which was significantly higher in bead injected and ONC RGC layers compared to controls. Thus, these results demonstrated common phenotypes associated with glaucomatous injury including glial activation and apoptosis, and further validate the use of these models to study the glaucomatous loss of RGCs.

### Chronic and acute injured retinas exhibit differential loss of RGC subtypes

RGCs are a highly diverse population of neurons which serve the common purpose of transmitting visual information to the brain. Until recently, the separation of RGCs into specific subtypes was achieved by morphological and functional studies, with each type of RGC exhibiting various dendritic shapes, sizes, and stratification as well as different physiological response to visual information. The heterogeneous nature of RGCs has become increasingly more apparent in recent years with the discovery of novel molecular markers which identify specific populations of subtypes. RGC subtypes have been classified into a few major groups which can be further divided into more than 40 subtypes based on the combinatorial expression of different molecular markers^7–9^.

**Figure 3:**
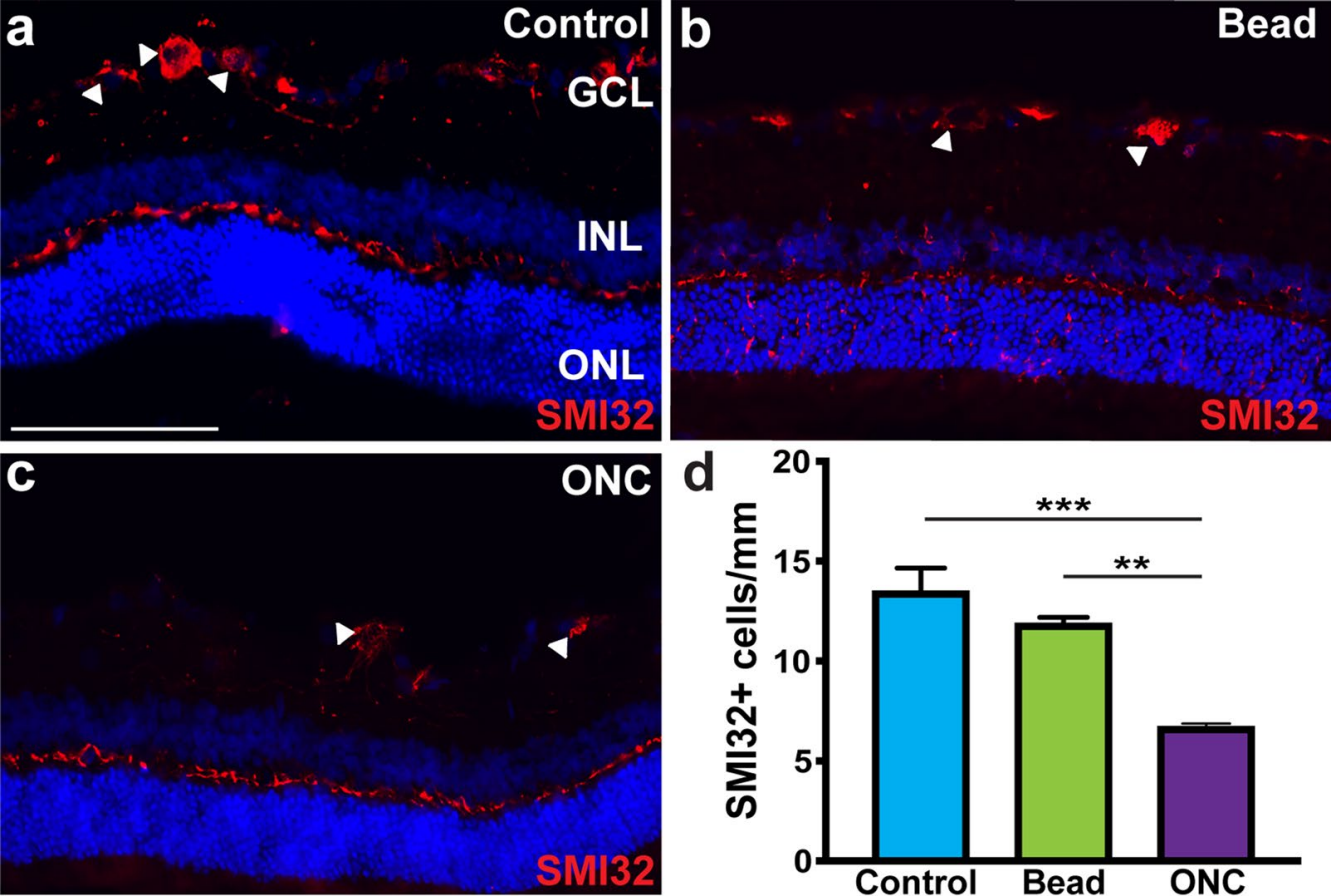
Alpha-RGCs display susceptibility to injury. (**a**) Alpha-RGCs were identified within the ganglion cell layer by the expression of SMI32. (**b**–**d**) Following ONC, the expression of SMI32 was significantly decreased compared to both controls and the bead occlusion model. Error bars represent S.E.M. Scale bars equal 100 µm. Statistical differences were determined using a One-way ANOVA followed by Tukey’s post hoc with 95% confidence. (**p < 0.01, ***p < 0.001). n = 6 retinas were analyzed per group, with 4 cross sections and numerous technical replicates per cross section. Cross sections contained both nasal and temporal parts, with technical replicates including areas from both the central and peripheral retina.

To explore the susceptibility of these RGC subtypes to glaucomatous injury, initial efforts were first directed toward the analysis of alpha-RGCs, a group of cells that have various responses to light and dark stimuli and can be further divided into 3 subsets; ON, Sustained OFF, and Transient OFF based on the combinatorial expression of different molecular markers^39–42^. Osteopontin and SMI32 have been established as pan-identifiers of alpha-RGCs, and as such SMI32 expression was utilized to visualize and quantify this class of cells. Immunocytochemical analyses demonstrated the expression of SMI32 in cells selectively within the ganglion cell layer of control retinas (Fig. 3). Following acute ONC injury, a significant decrease in the average number of SMI32-positive alpha-RGCs was identified in the RGC layer, although no decrease in alpha-RGCs in the chronic bead occlusion model was observed compared to controls. These results suggest a heightened susceptibility of alpha-RGCs following ONC injury.

**Figure 4:**
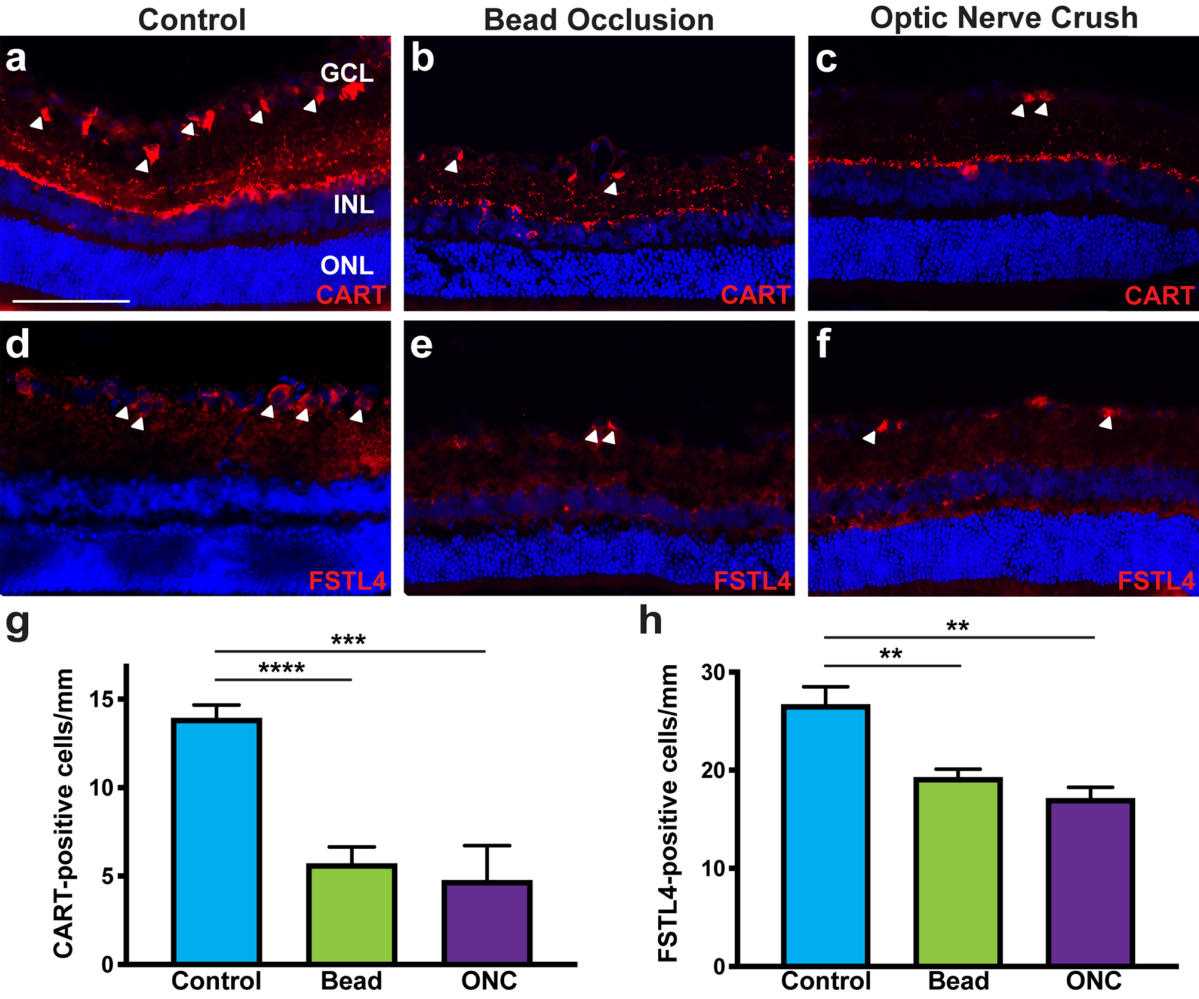
Chronic bead injection and acute ONC injuries result in a severe loss of direction selective-RGCs. (**a**–**c**, **g**) CART expression identified ON–OFF direction selective-RGCs in rat retinas and was significantly decreased in bead injected and ONC retinas. (**d**–**f**, h) A subset of ON direction selective-RGCs were identified by FSTL4, with positive cell bodies found exclusively within the ganglion cell layer. Following bead injection or ONC, FSTL4 expression was significantly reduced. Error bars represent S.E.M. Scale bars equal 100 µm. Statistical differences were determined using a One-way ANOVA followed by Tukey’s post hoc with 95% confidence. (**p < 0.01, ***p < 0.001, ****p < 0.0001). n = 6 retinas were analyzed per group, with 4 cross sections and numerous technical replicates per cross section. Cross sections contained both nasal and temporal parts, with technical replicates including areas from both the central and peripheral retina.

Direction selective-RGCs have the unique ability to respond to preferred directional motion in response to light and dark stimuli^43,44^ and thus, effects of chronic and acute injuries were also investigated upon these cells. Numerous molecular markers have been established to identify these cells, with the combinatorial expression of various markers used to characterize specific subsets of direction selective-RGCs^8,10,45,46^. Two types of direction selective-RGCs were investigated in this study, ON–OFF direction selective-RGCs identified by CART expression, and a subset of ON direction selective-RGCs identified by FSTL4 expression. CART and FSTL4 were highly expressed within the ganglion cell layer of control retinas (Fig. 4). When analyzed after injury, the expression of both direction selective-RGC markers was significantly decreased in a similar fashion compared to controls. Thus, direction-selective RGCs also demonstrated a high susceptibility to both acute and chronic injuries.

**Figure 5:**
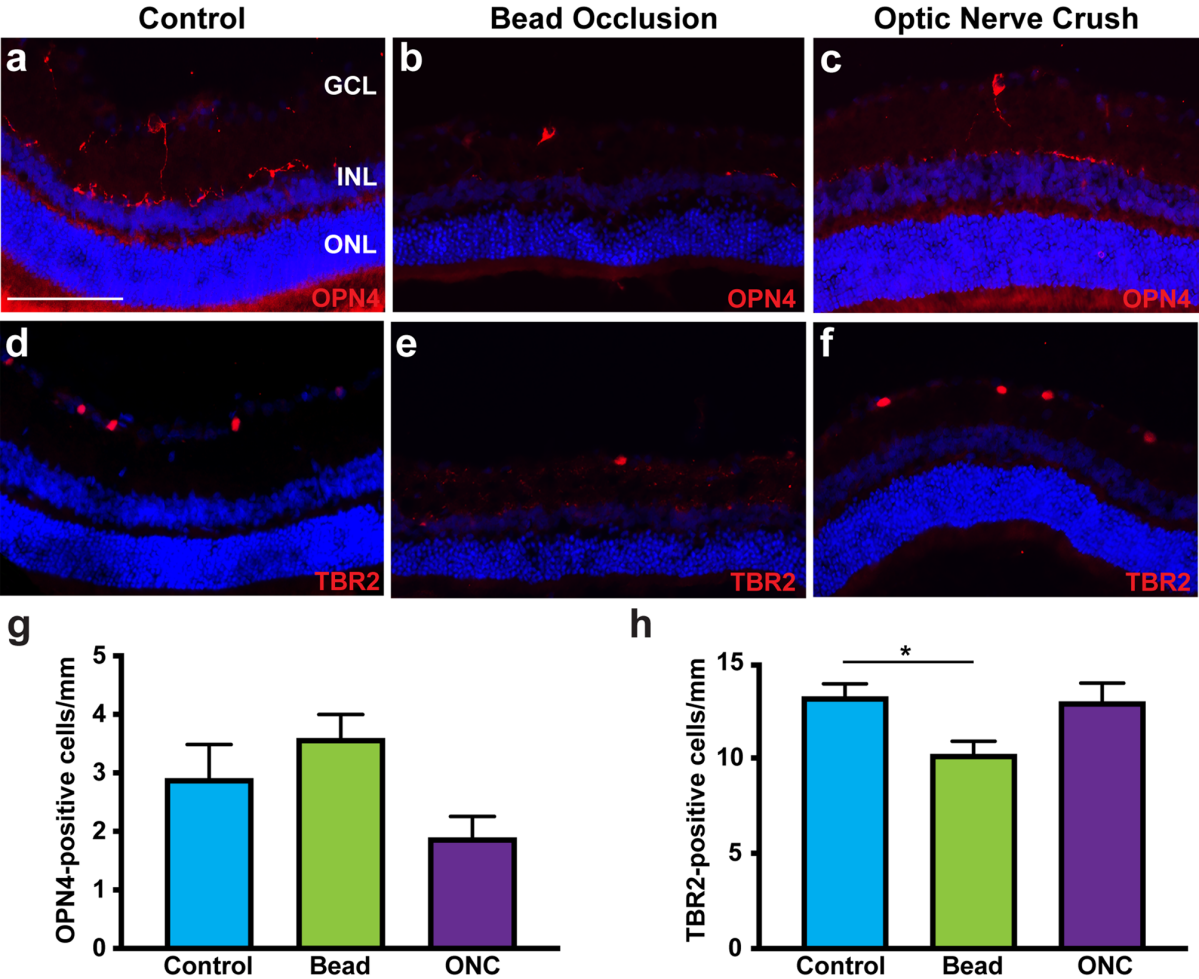
ipRGCs demonstrated differential survival in response injury. (**a**–**c**, **g**) OPN4 expression was observed in similar levels in control, bead injected and ONC retinas, indicating a preferential survival of these cells in response to injury. (**d**–-**f**, **h**) TBR2-expressing ipRGCs were significantly decreased in bead injected retinas, but not ONC retinas, compared to controls. Error bars represent S.E.M. Scale bars equal 100 µm. Statistical differences were determined using a One-way ANOVA followed by Tukey’s post hoc with 95% confidence (*p < 0.05). n = 6 retinas were analyzed per group, with 4 cross sections and numerous technical replicates per cross section. Cross sections contained both nasal and temporal parts, with technical replicates including areas from both the central and peripheral retina.

Lastly, the loss of ipRGCs was explored within both chronic and acute models of retinal injury. This unique class of RGCs is characterized by the distinct ability to respond directly to light stimuli, aiding in the formation of circadian rhythms and pupillary reflexes^47–52^. In this study, ipRGCs were readily identified by the expression of OPN4 and TBR2 within the ganglion cell layer (Fig. 5). Following bead injection or ONC, no significant changes were detected in the average number of OPN4-expressing ipRGCs, although the number of TBR2-expressing cells was significantly decreased only in the bead injected retinas. These results suggest the ability for ipRGCs to exhibit a degree of resilience to these glaucomatous injuries.

Taken together, experiments conducted in the current study compared the effects of the chronic bead injection and acute ONC injury models upon the degeneration of RGCs, including different RGC subtypes. More so, results of this study elucidated a differential loss and susceptibility of major RGC subtypes that varied between injury models.

## Discussion

RGCs are the main cell type affected in optic neuropathies, with degeneration of these cells leading to profound phenotypes and eventual loss of vision. Such diseases are rapidly increasing in their prevalence worldwide, with a projected 111.8 million people affected by 2040^53^. Therefore, a need exists to develop new treatments and therapeutics targeted at specific disease mechanisms causing the degeneration of RGCs. Previous studies have elucidated the diverse nature of RGCs, with numerous subtypes discovered to date with differing morphological, functional, and molecular features^7,8^. More so, recent studies have also suggested a differential susceptibility of RGC subtypes in disease or injury states^9,12–25^, providing new opportunities for targeting specific subtypes for treatment and therapeutics. Efforts of the current study utilized two model systems, including chronic bead occlusion and acute ONC injury models, to elucidate the degeneration of RGCs. Results demonstrated a significant degeneration of RGCs and a variety of other disease-associated phenotypes, including glial activation and apoptosis. More so, the investigation of major RGC subtypes following injury indicated a heightened susceptibility of direction selective-RGCs and alpha-RGCs, while ipRGCs displayed a preferential survival compared to those other subtypes.

The most widely studied phenotype associated with disease and injury to the retina is profound degeneration and loss of RGCs^2,54^. Following bead injection or ONC injury, retinas displayed a significant loss of RBPMS-expressing RGCs as well as a significant decrease in the number of nuclei within the ganglion cell layer, indicative of retinal thinning. More so, both models resulted in presumptive injury responses including reactive gliosis, microglial activation and infiltration, and increased apoptosis, all of which are characteristics associated with disease progression observed in optic neuropathies^31,33,36^. These results demonstrated the effectiveness of both the bead occlusion and ONC models to serve as effective models of RGC degeneration.

Previous studies have used various injury and disease models in different animals to elucidate the loss of specific RGCs subtypes, with results somewhat variable based on the model system or the organism used^12–20^. Results of the current study demonstrated a high susceptibility for direction selective-RGCs and alpha-RGCs, with this phenomenon observed similarly in a number of other studies^22,23,25^. As such, the loss of these two subtypes in our study validates their susceptibility to disease and sets the precedence for future studies to elucidate underlying disease mechanisms causing their degeneration. Furthermore, future studies should also be focused on how to target direction selective-RGCs and alpha-RGCs for therapeutics and cell replacement strategies.

In contrast to the high susceptibility of certain RGC subtypes, non-image forming ipRGCs exhibited differential survival following injury compared to image forming direction selective- and alpha-RGCs in the current study. The preferential survival of ipRGCs has been observed in other studies which utilized rat and mouse injury and disease models, with this subtype in particular demonstrating resilience to insult^9,13,14,22,24,25,55–58^. ipRGCs are unique in that they have the ability to respond to light using the photopigment melanopsin, and play a role in non-image forming functions including circadian rhythms and pupillary reflexes by projecting to the suprachiasmatic nucleus, a non-image forming brain region^47,49,50^. Due to the unique nature of their functionality, this may lead to their resilience to insult as they are not necessary for image formation during visual transduction.

In the current study, ONC was implemented as a model of RGC loss and degeneration, with physiological relevance to acute injuries such as traumatic optic neuropathies^18,59^. Conversely, the bead occlusion model mimics the consistent high intraocular pressure observed in chronic forms of glaucoma^16,30^. This model differs from the ONC model as it provides a more physiologically-relevant representation of glaucomatous disease manifestation through the progressive increase in pressure resulting in the degeneration of RGCs over time. This study utilized the ONC injury as well as the bead occlusion model in order to provide a well-rounded examination of RGC degeneration and RGC subtype specific loss following injury and disease. In future studies, it will be important to examine these differences in primate retinas, as recent studies have demonstrated disparities in the subtypes of rodent retinas compared to macaque retinas^60^.

As optic neuropathies have a sizable impact on the quality of life for patients, a need exists for the development of targeted therapeutics to aid patients experiencing such diseases. As this study and others have shown, RGC subtypes exhibit differential survival in response to disease and injury, with the future ability to study mechanisms underlying differential RGC-subtype loss. Furthermore, the opportunity also exists to develop more targeted treatments for disease phenotypes, including the selective neuroprotection of highly susceptible subtypes as a potentially novel therapeutic strategy for these degenerating RGCs.

## Supplementary information

Supplementary Information
