# Supplementary material for: Differential susceptibility of retinal ganglion cell subtypes in acute and chronic models of injury and disease
Source: Sci Rep. 2020 Oct 15;10:17359. doi: 10.1038/s41598-020-71460-6 (PMC7566630; doi:10.1038/s41598-020-71460-6)
Supplement: Supplementary file 1 — Supplementary Information [file 41598_2020_71460_MOESM1_ESM.docx]

**Title**

Differential Susceptibility of Retinal Ganglion Cell Subtypes in Acute and Chronic Models of Injury and Disease

**Authors/Affiliations**

Kirstin B. VanderWall^1^, Bin Lu^2^, Jorge S. Alfaro^2^, Anna R. Allsop^1^, Alexa S. Carr^1^, Shaomei Wang^2^, Jason S. Meyer^3-5^

^1^Department of Biology, Indiana University Purdue University Indianapolis, Indianapolis IN 46202 USA

^2^Department of Biomedical Sciences, Regenerative Medicine Institute, Cedars-Sinai Medical Center, Los Angeles CA 90048 USA

^3^Department of Medical and Molecular Genetics, Indiana University School of Medicine, Indianapolis IN 46202 USA

^4^Deparment of Ophthalmology, Glick Eye Institute, Indiana University School of Medicine, Indianapolis IN 46202

^5^Stark Neurosciences Research Institute, Indiana University School of Medicine, Indianapolis IN 46202 USA

**Contact**

meyerjas@iu.edu

Shaomei.Wang@cshs.org

| **Supplemental Table 1**: Primary Antibody Information | | | |
| --- | --- | --- | --- |
| Primary Antibodies | Company | Catalog Number | Dilution |
| CART | Phoenix Pharmaceuticals | H-003-62 | 1:1000 |
| Caspase-3, active | Promega | G7481 | 1:200 |
| FSTL4 | Novus | 91913 | 1:500 |
| GFAP | Millipore | MAB360 | 1:200 |
| IBA1 | Abcam | Ab5076 | 1:200 |
| OPN4 | ThermoScientific | PA1-781 | 1:500 |
| RBPMS | PhosphoSolutions | 1830-RBPMS | 1:500 |
| SMI-32 | Calbiochem Millipore | NE1023 | 1:500 |
| TBR2 | Abcam | ab23345 | 1:500 |

**Supplemental Table 1:** The table represents the primary antibodies used throughout the study including the catalog number and their respective dilutions.


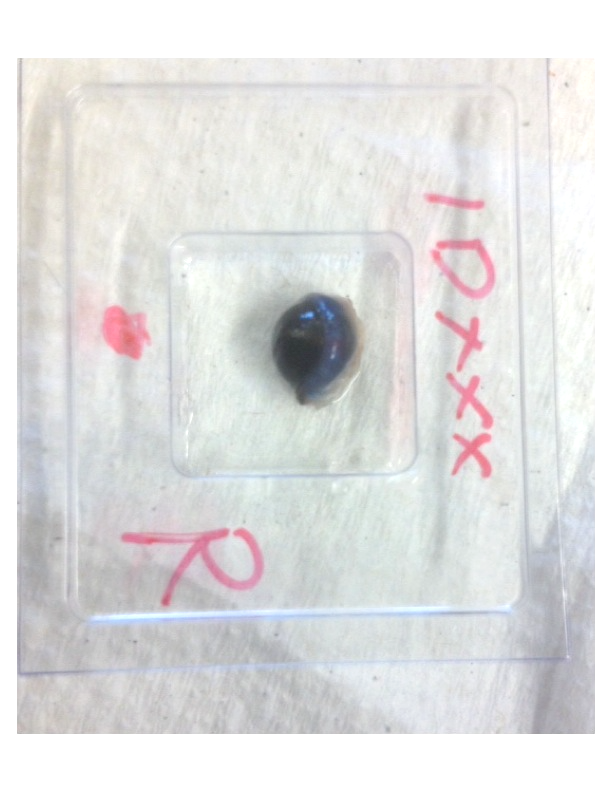


Dorsal

Nasal

Temporal

Ventral

6

3

12

9

11

1

11

16

1

6

11

5

15

20

5

10

…

…

11

10

35

40

25

30

**Supplemental Figure 1**: Methods for cryosectioning retinas. The eyeball was placed in an embedding mold and orientation was noted with the dorsal present at 12 o’clock. Starting from the dorsal edge, 10µm horizontal sections were cut containing both nasal and temporal parts in a series of 5 with 4 sections per slide.

**Supplemental Figure 2. Microbeads-induced elevated IOP.** Changes in intra-ocular pressure between treated and untreated rats 1, 4, and 5 weeks post injections (W1, W4, W5, respectively)(n=9, **p<0.1, ***p<0.001).
